# Comparison of Simoa and Lumipulse Neurofilament Light Chain Measurements in Alzheimer’s Cerebrospinal Fluid: Preliminary Findings

**DOI:** 10.3390/brainsci15090911

**Published:** 2025-08-24

**Authors:** Silvia Boschi, Alberto Mario Chiarandon, Aurora Cermelli, Chiara Lombardo, Giulia Gioiello, Giulia Montesano, Elisa Rubino, Giulio Mengozzi, Innocenzo Rainero, Fausto Roveta

**Affiliations:** 1 Department of Neuroscience and Mental Health, AOU Città della Salute e della Scienza di Torino, 10126 Torino, Italy; silvia.boschi@unito.it (S.B.); alberto.chiarandon@unito.it (A.M.C.); aurora.cermelli@unito.it (A.C.); c.lombardo@unito.it (C.L.); elisa.rubino@unito.it (E.R.); innocenzo.rainero@unito.it (I.R.); 2 Department of Medical Sciences, University of Torino, 10126 Torino, Italy; giulia.gioiello@unito.it (G.G.); giulia.montesano@unito.it (G.M.); giulio.mengozzi@unito.it (G.M.); 3 Clinical Biochemistry Laboratory “Baldi e Riberi”, AOU Città della Salute e della Scienza di Torino, 10126 Torino, Italy; 4 Aging Brain and Memory Clinic, Department of Neuroscience “Rita Levi- Montalcini”, University of Torino, 10126 Torino, Italy

**Keywords:** Alzheimer’s disease, neurofilament light chain, Simoa, Lumipulse, cerebrospinal fluid, mild cognitive impairment, biomarkers

## Abstract

**Background:** Neurofilament light chain (NfL) is a promising biomarker of neuroaxonal injury, increasingly used to monitor neurodegeneration in Alzheimer’s disease (AD). Multiple analytical platforms are available for NfL quantification in cerebrospinal fluid (CSF), but data on cross-platform consistency remain limited. **Objective:** This pilot study aimed to provide CSF NfL concentrations measured using Simoa and Lumipulse immunoassays in patients with biologically confirmed AD. **Methods:** Twenty-eight patients with cognitive impairment fulfilling the biological criteria for AD were enrolled. CSF NfL levels were measured using both Simoa and Lumipulse immunoassays. Statistical analyses assessed intra-individual agreement, correlation between platforms, and associations with cognitive status. **Results:** NfL concentrations measured with Simoa and Lumipulse showed a strong positive correlation between platforms (Spearman’s ρ = 0.965, *p* < 0.001), demonstrating excellent analytical concordance. **Conclusions:** In this pilot study, Simoa and Lumipulse yielded strongly correlated CSF NfL measurements, providing initial evidence of cross-platform consistency. However, these findings require confirmation in larger and diverse cohorts before definitive validation.

## 1. Introduction

Alzheimer’s disease (AD) is the most prevalent neurodegenerative disorder globally, representing the leading cause of dementia in elderly populations [1]. Clinically, AD is characterized by a gradual and progressive decline in cognitive function, especially memory, along with impairments in language, executive function, and visuospatial abilities. These symptoms are underpinned by neuropathological changes, including extracellular amyloid-beta (Aβ) plaques, intracellular neurofibrillary tangles composed of hyperphosphorylated tau protein, synaptic dysfunction, and ultimately widespread neuronal loss [2]. As the disease advances, affected individuals progressively lose independence, often requiring full-time care, which results in a substantial socio-economic burden on caregivers and healthcare systems [3].

In recent years, advances in the understanding of AD pathophysiology have prompted a shift from purely clinical to biologically based diagnostic frameworks [4]. This evolution has placed CSF biomarkers at the core of diagnostic and research criteria. CSF biomarkers reflect key pathological events and now serve as objective measures to detect Alzheimer’s pathology in vivo [5]. The “core” biomarkers for AD include decreased CSF Aβ42, indicating cerebral amyloid deposition, and increased total tau (t-tau) and phosphorylated tau (p-tau), representing neurofibrillary pathology and neuronal injury, respectively [6].

In addition to these core biomarkers, other molecular targets have been investigated to further improve diagnostic precision and disease monitoring. For instance, Xia et al. developed a specific enzyme-linked immunosorbent assay to detect beta-amyloid protein oligomers in plasma and brain tissue, demonstrating the feasibility of detecting soluble Aβ aggregates, which are believed to be the most neurotoxic species [7]. Such oligomeric forms may provide earlier or complementary information compared to Aβ42 levels alone, and their measurement is gaining interest in both fluid biomarker and imaging research.

However, while tau-related biomarkers are informative, they do not fully capture the extent of axonal damage occurring in the disease. In this context, NfL has emerged as an additional, sensitive biomarker for neuroaxonal damage [8]. NfL is a cytoskeletal protein found predominantly in large, myelinated axons and serves a structural role by contributing to axonal stability, diameter, and conduction velocity [9]. It is the most abundant and soluble of the neurofilament subunits, and upon axonal injury, NfL is released into the interstitial fluid, from where it enters the CSF and, eventually, the bloodstream [10]. The small size of the molecule (~68 kDa) facilitates its diffusion, and its levels in CSF and plasma have been shown to correlate with the degree of axonal degeneration [11,12,13]. In AD, elevated CSF NfL levels have been associated with disease severity, brain atrophy on imaging, and faster cognitive decline [14,15]. Importantly, NfL increases early in the disease course, even during the preclinical or prodromal stages, and may thus serve as a marker of ongoing neurodegeneration when combined with amyloid and tau pathology indicators.

The detection and quantification of NfL require highly sensitive and specific immunoassays, due to the low physiological concentrations of the protein and its relevance across various neurodegenerative diseases. One of the most widely adopted platforms for NfL quantification is the Single Molecule Array (Simoa), a digital immunoassay technology developed by Quanterix (Billerica, MA, USA) [16]. Simoa operates on the principle of single-molecule detection using femtoliter-sized wells, which enable the capture and quantification of individual enzyme-labeled immune complexes. The assay follows a traditional sandwich format, with magnetic beads coated with a capture antibody against NfL, and a detection antibody labeled with an enzyme. The beads are isolated into individual wells, where enzymatic reactions generate fluorescent signals counted digitally to produce ultra-sensitive readouts, capable of detecting proteins at subfemtomolar concentrations [17]. The Simoa platform supports multiplexing and offers a broad dynamic range, making it highly suitable for research applications. Nevertheless, the platform requires specialized equipment, trained personnel, and considerable cost, which can limit its immediate scalability in routine clinical laboratories [18].

Alternatively, the Lumipulse^®^ platform, developed by Fujirebio (Ghent, Belgium), is a fully automated chemiluminescent enzyme immunoassay (CLEIA) system that allows for high-throughput quantification of various biomarkers, including NfL. The assay uses a two-step sandwich technique with antigen capture on magnetic particles and signal generation via a chemiluminescent reaction. While its analytical sensitivity is slightly lower than that of Simoa, the platform offers substantial advantages in terms of automation, shorter run times, and compatibility with clinical laboratory infrastructure [19,20]. Lumipulse assays are CE-marked for in vitro diagnostic use and have been validated for use with a variety of CSF and plasma biomarkers relevant to Alzheimer’s disease, such as Aβ42, Aβ40, t-tau, and p-tau181 [21,22]. Beyond technical considerations, the clinical utility of NfL is increasingly well-established. In Alzheimer’s disease, CSF NfL levels correlate with cognitive impairment severity, rate of decline, and structural brain changes observable on MRI, including cortical thinning and ventricular enlargement [1,23]. NfL concentrations have also been shown to predict progression from mild cognitive impairment (MCI) to dementia and are elevated in preclinical stages, making them useful for early identification of individuals at risk [24]. Importantly, when used alongside classical AD biomarkers, NfL provides complementary information about the intensity of ongoing neurodegeneration, rather than the underlying amyloid or tau pathology. This combined biomarker approach has the potential to improve diagnostic precision and stratify patients based on both pathological burden and neurodegenerative activity [25]. NfL is also increasingly explored as a pharmacodynamic biomarker in clinical trials of disease-modifying therapies. Changes in CSF or plasma NfL levels may reflect therapeutic efficacy in slowing neuronal damage, particularly in trials involving anti-amyloid or anti-tau agents [26]. The availability of reliable, scalable assays like Lumipulse is therefore critical to translate these advances into real-world clinical monitoring. Additionally, NfL levels may aid in the differential diagnosis of AD versus other neurodegenerative conditions. For instance, CSF NfL concentrations tend to be higher in frontotemporal dementia, amyotrophic lateral sclerosis, and prion diseases than in AD, reflecting the more aggressive nature of axonal degeneration in these disorders [27]. Although not disease-specific, NfL remains a sensitive biomarker of neuroaxonal injury and can enhance diagnostic confidence when interpreted within the broader clinical and biomarker context. In summary, the measurement of NfL in CSF represents a valuable tool for the assessment of neurodegeneration in Alzheimer’s disease. The choice of assay platform plays a pivotal role in ensuring the reliability and interpretability of results. While the Simoa platform provides unmatched sensitivity suitable for research environments, the Lumipulse platform offers automation, clinical applicability, and robust performance for routine use. Given the growing role of NfL in both diagnostics and therapeutic monitoring, it is essential to validate the concordance between these two technologies [28]. The present study aims to directly compare CSF NfL concentrations measured using Simoa and Lumipulse platforms in patients with biomarker-confirmed Alzheimer’s disease. By evaluating analytical agreement, variability, and diagnostic relevance, this comparison seeks to inform assay standardization and support the broader clinical adoption of NfL as a routine biomarker.

## 2. Materials and Methods

### 2.1. Subjects

In this study, 28 patients with cognitive impairment referred to the Aging Brain and Memory Clinic at the University Hospital “Città della Salute e della Scienza” of Torino, Italy from May 2023 and January 2025 were selected. All participants underwent CSF analysis, and only patients fulfilling the ATN-based biological criteria for Alzheimer’s disease, as defined by the 2018 NIA-AA framework, were included in the study [4].

Patients underwent a comprehensive diagnostic evaluation, including neurological examination, cognitive testing, and brain neuroimaging, as previously described [29,30]. Based on the Clinical Dementia Rating (CDR) scale, patients were classified into two groups: MCI due to AD (MCI, CDR = 0.5), and AD dementia (CDR ≥ 1). No patients with non-AD pathologies or suspected mixed etiologies were included in the study.

The study was approved by the local ethics committee (Comitato Etico Interaziendale, AOU Città della Salute e della Scienza di Torino, protocol number 0143406/2022) and all patients provided written informed consent, in accordance with the Declaration of Helsinki.

### 2.2. Biological Sample Collection and Processing

CSF and blood samples were collected on the same day under standardized conditions [31]. Lumbar puncture was performed after overnight fasting. CSF was collected in polypropylene tubes, centrifuged at 2000× *g* for 10 min at 4 °C within one hour of collection, and aliquoted into polypropylene vials before storage at −80 °C. DNA was extracted from whole blood, and APOE genotyping was performed using standard protocols [32].

All core AD biomarkers reported in Table 1, including Aβ42, Aβ40, total Tau, and phosphorylated Tau (pTau181), were measured using the Lumipulse G1200II automated system (Fujirebio, Ghent, Belgium) following the manufacturer’s instructions and in accordance with previously established procedures [18].

### 2.3. NfL Quantification

NfL concentrations in cerebrospinal fluid were measured using two distinct immunoassay platforms: the Simoa and the Lumipulse automated chemiluminescent enzyme immunoassay (CLEIA) system.

Simoa is a digital immunoassay platform developed by Quanterix (Billerica, MA, USA) that enables ultra-sensitive detection of proteins at the single-molecule level by isolating enzyme-labeled immune complexes in thousands of femtoliter-sized wells. It is widely used in research for its high sensitivity and precision, particularly for low-abundance biomarkers such as NfL. The first was the Simoa™ Neurology 2-Plex B assay kit (NfL; Catalog #103520, Lot Number 504401, Quanterix, Billerica, MA, USA), analyzed on the SR-X Biomarker Detection System. CSF samples were diluted 1:40, randomly distributed across 96-well plates, and measured in a blinded manner. Quality control samples met predefined performance criteria, with inter-plate coefficients of variation below 10%.

The second quantification was conducted using the Lumipulse G1200II automated system (Fujirebio, Ghent, Belgium), based on chemiluminescent enzyme immunoassay (CLEIA) technology. The Lumipulse platform was developed to allow automated, high-throughput analysis of clinical biomarkers with good sensitivity and reproducibility. It is commonly used in routine diagnostics and has been CE-marked for several Alzheimer’s disease biomarkers, including Aβ42, t-tau, and p-tau. The Lumipulse G NfL assay (ref. 231456) was used according to the manufacturer’s instructions. All samples were analyzed in duplicate, and intra-assay coefficients of variation were consistently below 10%. NfL values were expressed in pg/mL for both assays.

### 2.4. Statistical Analysis

Statistical analyses were performed using Jamovi (version 2.3.28) and R (version 4.5.0). Continuous variables were expressed as mean and standard deviation (SD) and as median and interquartile range (IQR). Normality was assessed using the Shapiro–Wilk test.

Group comparisons between patients with MCI and those with dementia were carried out using parametric or non-parametric tests, depending on the nature and distribution of the variables. Specifically, Student’s *t*-test or Welch’s *t*-test was used for continuous variables with normal distribution, while the Mann–Whitney U test was applied for non-normally distributed data. For categorical variables, Chi-squared or Welch-corrected tests were used as appropriate. Paired comparisons of NfL concentrations obtained using the two analytical platforms (Lumipulse and Simoa) were performed using the Wilcoxon signed-rank test. To explore associations between demographic variables, CSF biomarkers, and NfL values measured with both methods, Spearman’s rank correlation coefficient (ρ) was used. To address the sex imbalance, we computed sex-stratified Spearman correlations between platforms.

We assessed agreement using Bland–Altman plots (primary on the log scale, secondary on the original pg/mL scale), estimating mean bias and 95% limits of agreement and testing proportional bias via linear regression of differences on means. Overall concordance was summarized with Lin’s CCC (95% CI).

All statistical tests were two-tailed, and a *p*-value < 0.05 was considered statistically significant. No correction for multiple comparisons was applied due to the exploratory nature of the study.

## 3. Results

### 3.1. Overall Population

A total of 28 patients were included in the study, of whom 18 were diagnosed with MCI and 10 with Dementia (Table 1). In the MCI group, the mean age at sampling was 70.7 ± 5.52 years (median = 72.0, IQR = 67.5–75), while in the dementia group it was 70.7 ± 10.7 years (median = 73.5, IQR = 69.5–77.5).

The average number of years of education was 12.1 ± 3.82 (median = 12.0, IQR = 11.0–13.0) in the MCI group and 11.4 ± 3.84 (median = 11.0, IQR = 8.5–13.8) in the dementia group.

Females represented 58.3% of the overall study participants. In sex-stratified analyses, the Spearman correlation remained similar within each subgroup (Female: r = 0.984, n = 16; Male: r = 0.971, n = 12).

The APOE genotype was available for 24 participants: 16 in the MCI group and 8 in the dementia group. Among those with MCI, 9 individuals (56.25%) were carriers of at least one ε4 allele, whereas in the dementia group, 5 individuals (62.5%) carried at least one ε4 allele. Overall, 14 participants (57.1%) were ε4 carriers. To evaluate whether APOE genotype influenced the correlation between the two measurement platforms, we performed a partial correlation analysis controlling for APOE status. The correlation between CSF NfL levels measured by Simoa and Lumipulse remained high and statistically significant (r = 0.987, *p* < 0.0001, n = 24), indicating that the observed concordance is not explained by the APOE genotype.

### 3.2. CSF NfL Profile

NfL concentrations were measured in cerebrospinal fluid using both the Simoa and Lumipulse platforms. In the MCI group, the mean NfL levels were 1044 ± 602 pg/mL (median = 928, IQR = 627–1219) with the Lumipulse assay, and 1034 ± 598 pg/mL (median = 940, IQR = 636–1126) with the Simoa assay. In the dementia group, NfL concentrations averaged 1156 ± 500 pg/mL (median = 931, IQR = 915–1274) with Lumipulse, and 1208 ± 513 pg/mL (median = 1029, IQR = 863–1440) with Simoa.

**Figure 1:**
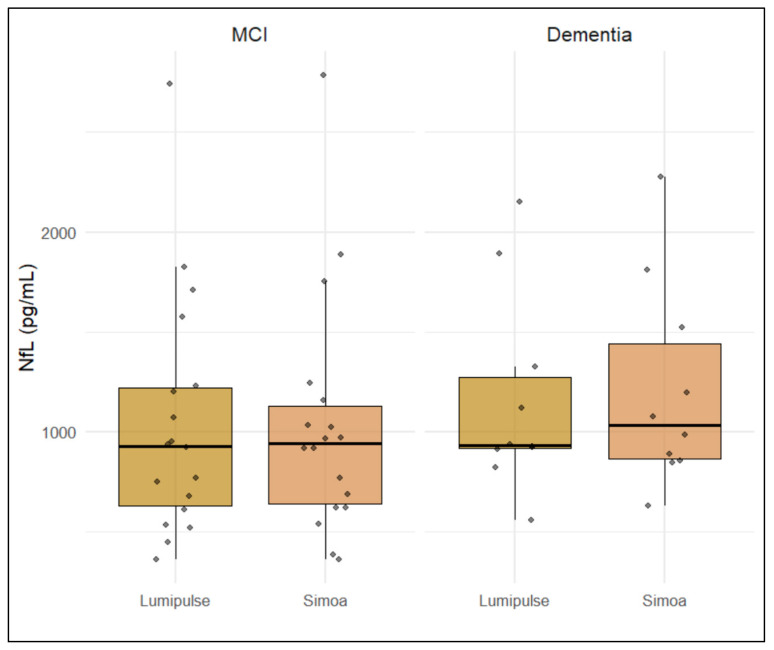
Boxplot showing CSF NfL concentrations measured with the Simoa and Lumipulse platforms across all participants (n = 28). The distribution of values reveals comparable medians and variability, with no statistically significant difference between the two methods (Wilcoxon signed-rank test, *p* = 0.316), supporting analytical concordance between the assays.

To assess the agreement between the two measurement platforms, a Wilcoxon signed-rank test was performed on paired NfL values from each patient, revealing no significant differences between methods (*p* = 0.316). This result is visually represented in the boxplot (Figure 1), which shows the distribution of NfL concentrations for both assays across all subjects. The boxplot illustrates comparable central tendencies and variability, further supporting the absence of systematic bias between the two techniques.

**Figure 2:**
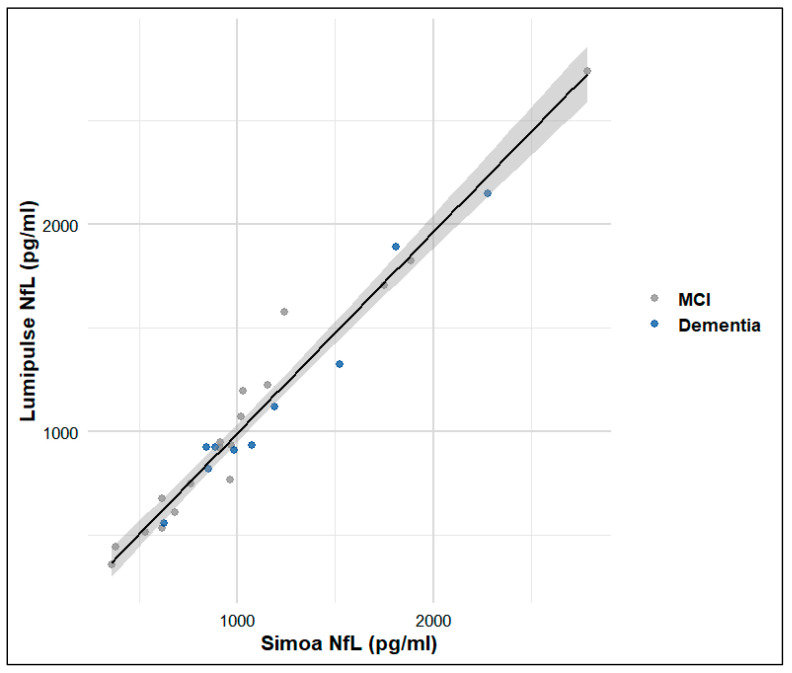
Scatterplot illustrating the correlation between CSF NfL concentrations measured by the two platforms. Each point represents an individual patient (n = 28). A strong positive correlation was observed (Spearman’s ρ = 0.965, *p* < 0.001), with data closely aligned along the diagonal, indicating high concordance between the two assays.

**Figure 3:**
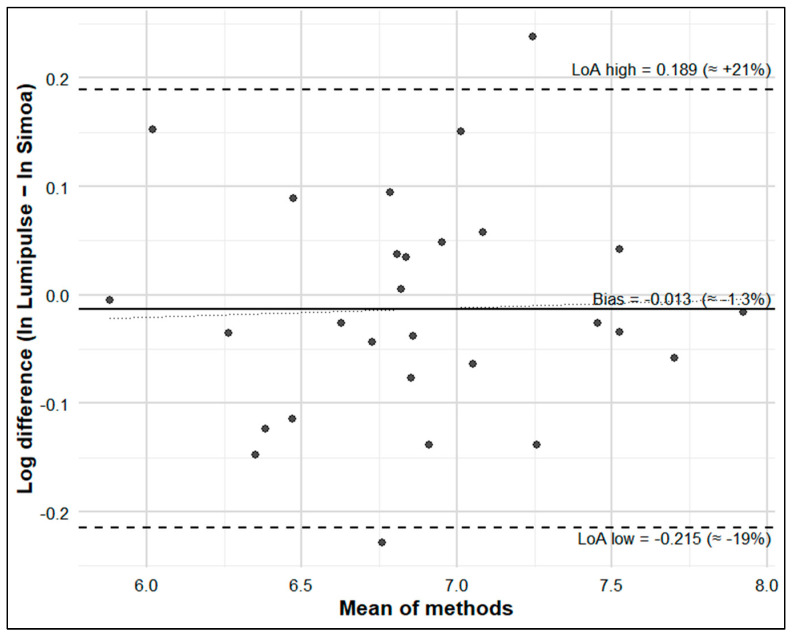
Bland–Altman comparison of Lumipulse vs. Simoa on the log scale (differences = ln Lumipulse − ln Simoa). The solid line shows the mean bias (≈−1.3%); dashed lines show the limits of agreement (≈−19% to +21%); the dotted regression line suggests no proportional bias.

Moreover, a near complete correlation was observed between Simoa and Lumipulse NfL levels (Spearman’s ρ = 0.965, *p* < 0.001), as depicted in the scatterplot (Figure 2). Agreement between Lumipulse and Simoa was high (CCC = 0.981; 95% CI 0.960–0.991). Bland–Altman showed a small mean bias on the log scale (~−1.3%) but wide limits of agreement (≈0.81–1.21; n = 28); on the linear scale, the mean difference was −11.8 pg/mL (95% CI −52.1 to 28.5) with LoA ranging from −225 to +201 pg/mL (Figure 3).

## 4. Discussion

In this study, we compared CSF neurofilament light chain levels measured using two analytical platforms Simoa and Lumipulse in a cohort of patients fulfilling the ATN criteria for AD. Our primary aim was to assess analytical agreement between these platforms in a within-subject design. Our findings demonstrate a strong correlation of concordance between the two methods, with no significant differences in absolute NfL concentrations and a near-complete positive correlation (Spearman’s ρ = 0.965). This suggests that both platforms may provide consistent results for CSF NfL quantification, although interchangeability cannot be definitively established with this limited samples size. These results support the preliminary analytical comparability of the two platforms and provide pilot evidence that NfL may serve as a reliable biomarker of neurodegeneration, pending further validation in larger cohorts.

The high correlation observed aligns with prior studies that have investigated cross-platform reliability for NfL quantification in CSF [33,34,35]. Despite inherent methodological differences, such as digital versus chemiluminescent signal detection, antibody selection, and calibration protocols, both assays provided consistent results across the clinical spectrum of cognitive impairment.

This consistency reinforces the feasibility of using NfL as a standardized biomarker in multicenter clinical and research settings.

In our cohort, patients with dementia showed numerically higher mean NfL levels than those with MCI, consistent with the notion that NfL reflects the intensity of ongoing neuroaxonal damage [36]. Although this difference did not reach statistical significance—likely due to the modest sample size and heterogeneity in disease severity, the observed trend remains clinically relevant and aligns with prior literature.

Importantly, the Lumipulse platform demonstrated excellent analytical performance while offering practical advantages in terms of automation, workflow integration, and clinical applicability. Given its CE-IVD status and compatibility with existing laboratory infrastructure, Lumipulse may represent a viable alternative to Simoa for routine use, especially in hospital-based diagnostic laboratories where scalability, cost, and ease of use are critical considerations [37]. Nevertheless, our findings should be interpreted with caution given the preliminary nature of the data and the methodological limitations discussed below.

Nonetheless, several limitations of this study should be acknowledged. First, the sample size was small, which may have limited the detection of subtle differences between subgroups and constrained statistical power.

Second, there was a significant gender imbalance between the MCI and dementia groups (*p* = 0.007). However, after a stratified correlation analyses, overall results were preserved.

Third, the lack of a healthy control group prevented assessment of platform comparability at the lower end of the NfL concentration range. While we focused on biologically confirmed AD to ensure a homogeneous diagnostic population, broader comparative cohorts including healthy controls will be essential in future studies. Moreover, validation in different biological samples (e.g., plasma) is warranted. Bland–Altman revealed near-zero bias but broad limits of agreement (≈0.81–1.21). Average concordance was strong, but single-patient interchangeability should be interpreted with caution and confirmed in larger cohorts. Overall, these limitations indicate that our results should be considered preliminary. They provide initial evidence of cross-platform consistency in CSF NfL measurement but require confirmation in larger, gender-balanced cohorts including patients of different etiologies and healthy controls.

## 5. Conclusions

Our study demonstrates a strong correlation between the Simoa and Lumipulse platforms in measuring cerebrospinal fluid NfL levels in patients across the Alzheimer’s disease continuum.

Although our results should be interpreted with caution and require confirmation in larger, independent cohorts, this study provides preliminary support for cross-platform consistency in CSF NfL measurement in Alzheimer’s disease.

## Figures and Tables

**Table 1:** Clinical and biomarker characteristics of subjects with Mild Cognitive Impairment (MCI) and dementia. Sex and APOE ε4 carrier status are expressed as percentages (%). All other variables are reported as mean ± standard deviation (SD). Group comparisons were assessed using appropriate statistical tests, and the corresponding *p*-values are shown in the rightmost column.

	MCI	Dementia	MCI	Dementia	*p*-Value
Age at sampling	N = 18	N = 10	70.7 ± 5.5	70.7 ± 10.7	0.993
Gender	N = 18	N = 10	7 (38.9%)	9 (90%)	0.007
APOE	N = 16	N = 8	9 (56.25%)	5 (62.5%)	0.781
CSF Aβ42 (pg/mL)	N = 18	N = 10	371 ± 131	362 ± 95	0.846
CSF ratio Aβ42/40	N = 17	N = 10	0.038 ± 0.008	0.042 ± 0.011	0.287
CSF t-tau (pg/mL)	N = 18	N = 10	676 ± 317	845 ± 377	0.252
CSF p-tau 181 (pg/mL)	N = 18	N = 10	103 ± 54	134 ± 69	0.289
CSF NfL Lumipulse (pg/mL)	N = 18	N = 10	1044 ± 602	1156 ± 500	0.606
CSF NfL Simoa (pg/mL)	N = 18	N = 10	1034 ± 598	1208 ± 513	0.426
Education	N = 18	N = 10	12.1 ± 3.8	11.4 ± 3.8	0.643
Mini-Mental State Examination	N = 18	N = 10	26.9 ± 1.8	15.1 ± 7.2	<0.001
Montreal Cognitive Assessment	N = 18	N = 5	20.7 ± 3.0	13.0 ± 4.9	0.020
Frontal Assessment Battery	N = 17	N = 8	13.5 ± 2.6	8.4 ± 3.0	0.001
Clock Drawing Test	N = 18	N = 8	11.5 ± 3.3	4.6 ± 3.7	<0.001

## Data Availability

The data are not publicly available due to privacy and ethical restrictions. Anonymized data not published within this article will be made available after reasonable request from any qualified investigator.

## Abbreviations

The following abbreviations are used in this manuscript:

Aβ	amyloid-beta plaques
NfL	neurofilament light chain
CSF	cerebrospinal fluid
AD	Alzheimer’s disease
t-tau	total tau
p-tau	phosphorylated tau
CLEIA	chemiluminescent enzyme immunoassay
MCI	mild cognitive impairment
